# Nomogram for predicting postoperative ileus after radical cystectomy and urinary diversion: a retrospective single-center study

**DOI:** 10.1080/07853890.2024.2329125

**Published:** 2024-03-18

**Authors:** Xiaoyu Sun, Chang Liu, Changwen Zhang, Zhihong Zhang

**Affiliations:** a Department of Urology, Tianjin Institute of Urology, The Second Hospital of Tianjin Medical University, Tianjin, China; b Department of Urology, Renmin Hospital of Wuhan Economic and Technological Development Zone (Hannan), Wuhan, China

**Keywords:** Postoperative ileus, bladder cancer, radical cystectomy, prediction model, nomogram

## Abstract

**Objective:**

To predict the incidence of postoperative ileus in bladder cancer patients after radical cystectomy.

**Methods:**

We retrospectively analyzed the perioperative data of 452 bladder cancer patients who underwent radical cystectomy with urinary diversion at the Second Hospital of Tianjin Medical University between 2016 and 2021. Univariate and multivariate logistic regression were used to identify the risk factors for postoperative ileus. Finally, a nomogram model was established and verified based on the independent risk factors.

**Results:**

Our study revealed that 96 patients (21.2%) developed postoperative ileus. Using multivariate logistic regression analysis, we found that the independent risk factors for postoperative ileus after radical cystectomy included age > 65.0 years, high or low body mass index, constipation, hypoalbuminemia, and operative time. We established a nomogram prediction model based on these independent risk factors. Validation by calibration curves, concordance index, and decision curve analysis showed a strong correlation between predicted and actual probabilities of occurrence.

**Conclusion:**

Our nomogram prediction model provides surgeons with a simple tool to predict the incidence of postoperative ileus in bladder cancer patients undergoing radical cystectomy.

## 1. Introduction

Bladder cancer is the most common type of urological tumour and can be subclassified as non-muscle invasive bladder cancer (NMIBC) and muscle invasive bladder cancer (MIBC). Radical cystectomy (RC) with urinary diversion is the standard curative treatment for MIBC without distant metastasis [1]. However, despite improvements in surgical technology and perioperative management, postoperative ileus (POI), a common postoperative gastrointestinal complication, is still an important clinical event after RC [2]. Clinical manifestations of POI include abdominal pain, nausea, vomiting, intolerance of diet and delayed passage of flatus or stool, and can lead to increased duration of hospitalization, medical expenses, and risk of downstream adverse events [3,4]. A recent nationwide study showed that 26.0% of patients who underwent RC developed POI [3]. Thus, in order to reduce the incidence of POI, it is essential to clarify the risk factors and identify the patients at greatest risk of POI. Although most of the previous studies on POI were based on gastrointestinal surgery, little is known about the risk factors for POI after RC and few predictive models have been established [5,6].

The nomogram model has been widely used in risk assessment and prognostic research for bladder cancer patients [7,8]. Instead of complex traditional regression models, nomograms can conduct convenient and accurate risk assessment for each patient by creating a user-friendly chart. This study aimed to investigate the perioperative risk factors for POI using large single-center data, then develop and validate a nomogram for patients who underwent RC.

## 2. Method

### 2.1. Study population

We retrospectively analyzed 452 bladder cancer patients who underwent RC with urinary diversion at the Second Hospital of Tianjin Medical University between January 2016 and December 2021. This study was approved by the hospital ethics committee and informed consent was obtained from each patient. Written consent was waived due to the retrospective nature of the study.

The inclusion criteria were as follows [1]: bladder cancer clinical stage T1-T4a [2]; no distant metastasis [3]; aged ≥18.0 years; and [4] scores ranging from I to III according to the American Society of Anaesthesiologists scale. The exclusion criteria were as follows [1]: patients did not meet the inclusion criteria [2]; palliative surgery; or [3] preoperative ileus. In order to better verify this model, we randomly divided the entire study population into training and validation samples at a ratio of 3: 1 (Table 1).

**Table 1.** Baseline characteristics of the study population.

Variable	Training sample(*n* = 339)	Validation Sample(*n* = 113)
Gender		
Male	275 (81.12%)	96 (84.96%)
Female	64 (18.88%)	17 (15.04%)
Age (mean ± SD)	60.230 ± 7.494	61.630 ± 7.019
Smoking	163 (48.08%)	51 (48.13%)
Hypertension	85 (25.07%)	29 (25.66%)
Diabetes mellitus	60 (17.70%)	19 (16.81%)
BMI (kg/m2)		
<18.5	21 (6.19%)	6 (5.31%)
18.5∼30	259 (76.41%)	89 (78.76%)
>30	59 (17.40%)	18 (15.93%)
Constipation	68 (20.06%)	25 (22.12%)
TURBt	151 (44.54%)	48 (42.48%)
Abdominal surgery	45 (13.27%)	14 (12.39%)
Chemical therapy	129 (38.05%)	37 (32.74%)
Anemia	59 (17.40%)	13 (11.50%)
Hypoalbuminemia	50 (14.75%)	17 (15.04%)
Renal inadequacy	27 (7.96%)	12 (10.62%)
surgical approach		
LRC	218 (64.31%)	77 (68.14%)
RARC	121 (35.69%)	36 (31.86%)
urinary diversion		
ileal conduit	257 (75.81%)	86 (76.11%)
orthotopic neobladder	82 (24.19%)	27 (23.89%)
PLND	191 (56.34%)	68 (60.18%)
Operative time (hours)	4.730 ± 0.860	4.800 ± 0.900
intestinal tract reconstruction time	40.343 ± 5.522	42.865 ± 5.564
Blood loss (milliliter)	373.600 ± 338.400	361.500 ± 268.510
Blood transfusion	131 (38.64%)	43 (38.05%)
pathological stage		
T1	23 (6.78%)	7 (6.19%)
T2	210 (61.95%)	78 (69.03%)
T3	81 (23.89%)	21 (18.59%)
T4	25 (7.37%)	7 (6.19%)

### 2.2. Outcome and variables

The outcome variable for the current study was POI occurring in the perioperative period. Despite its prevalence, the definition of POI varies among reports. Here, we defined POI as outlined in several highly cited papers [9,10]. Our definition included the following criteria [1]: Delayed or impaired bowel function after surgery, as evidenced by the presence of any of the following on postoperative day 5 or later: absence of gas or stool; intolerance of an oral diet after ingestion; diminished or absent bowel sounds; abdominal distension or abdominal pain [2]. the presence of multiple air-fluid levels that were confirmed radiologically. The occurrence of POI was independently diagnosed by two experienced surgeons.

The clinical and pathological data of valid patients were collected using the electronic medical records system. The recorded preoperative data included gender, age, body mass index (BMI), smoking, drinking, diabetes mellitus, hypertension, previous abdominal surgery, history of transurethral resection of bladder tumour (TURBt), intestinal tract reconstruction time, neoadjuvant chemotherapy, and preoperative levels of haemoglobin, albumin and creatinine. Relevant surgical data included different types of surgical approaches, methods of urinary diversion, estimated blood loss, blood transfusion, and pelvic lymph node dissection (PLND). Postoperative data included pathological TNM staging.

The surgical approaches used in our study included laparoscopic radical cystectomy (LRC) and robotic-assisted radical cystectomy (RARC). The most common methods of urinary diversion were ileal conduit, orthotopic neobladder and cutaneous ureterostomy. However, since cutaneous ureterostomy is the simplest form of urinary diversion normally associated with elderly and frail patients, its complication rate was significantly lower in patients compared with other methods [11,12]. Thus, to reduce selection bias, patients with cutaneous ureterostomy were not included in this study. The technology used for urinary diversion can be divided into intracorporeal and extracorporeal methods. Since a recent meta-analysis indicated that there were no significant differences between the two groups in POI [13]. We uses the extracorporeal method for LRC and the intracorporeal for RARC.

Enhanced Recovery after Surgery (ERAS) is a perioperative method for managing surgical patients. Currently, the standardized ERAS protocol and the impact on radical cystectomy remains understudied [14]. In our study, all patients were enrolled in an adapted ERAS protocol, which detailed items include patient education, optimization of bowel preparation, perioperative reduction of opioid use, prevention of intraoperative hypothermia, perioperative fluid management, early drinking and early ambulation.

### 2.3. Statistical analysis

Statistical analysis was performed using R version 4.0.1 (R Foundation for Statistical Computing, Vienna, Austria), GraphPad Prism 8.0 Software (GraphPad Software Inc., San Diego, CA, USA) and SPSS 26.0 software (SPSS Inc., Chicago, IL, USA). Continuous variables were described as mean ± standard deviation, and categorical variables were described as proportions. Student’s t-test and chi-square tests were used to confirm any statistical differences between continuous variables and categorical variables.

To identify the risk factors for POI, we first performed univariate logistic regression analysis. Variables that were significant with a *p* value < 0.20 were then included in a multivariate logistic regression analysis to screen for independent risk factors. Two- tailed P values < 0.05 were considered to be statistically significant. Forest plots were used to visualize the univariate and multivariate regression analyses data. Finally, the independent risk factors identified by multivariable logistic regression were used to build a nomogram model with R software.

Nomogram models were built and validated based on several recent studies [15]. The predictive ability of the model was evaluated with a receiver operating characteristic curve (ROC), while the consistency was verified using a calibration curve. Decision curve analysis (DCA) was adopted to determine the clinical usefulness and net benefit of the nomogram.

## 3. Results

### 3.1. Clinical status

A total of 452 bladder cancer patients were included in this study with POI occurring in 96 (21.2%) patients. The mean age of the total patient population was 61.14 ± 7.01 years, and 82.1% patients were male. 295 (65.3%) patients underwent LRC, while 157 (34.7%) underwent RARC. 343 (75.9%) patients underwent ileal conduit and 109 (24.1%) patients underwent orthotopic neobladder after RC. The mean operation time was 4.75 ± 0.87 h, and the mean estimated blood loss was 370.58 ± 322.11 ml. Based on the pathological TNM staging system for bladder cancer, there were 30 (6.6%) stage T1, 288 (63.7%) stage T2, 102 (22.6%) stage T3 and 32 (7.1%) T4a patients.

### 3.2. Risk factors associated with POI

**Figure 1.**
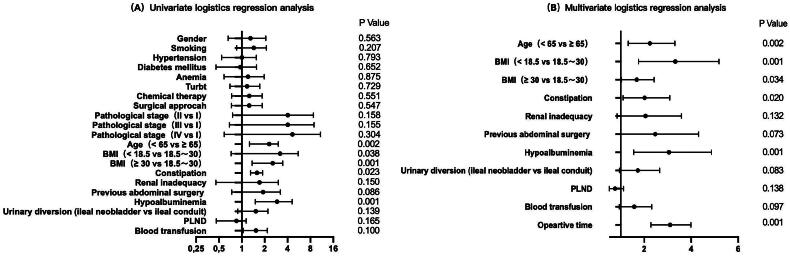
The forest plots show the results of univariate (A) and multivariate (B) analyses. In the multivariate logistic regression model, six independent risk factors for POI were further screened out, including age older than 65.0 years (OR =2.090, CI = 1.321–3.305, *p* = 0.002), low body mass index (OR =3.021, CI = 1.760–5.186, *p* = 0.001), high body mass index (OR =1.581, CI = 1.035–2.417, *p* = 0.034), Constipation (OR =1.843, CI = 1.101–3.088, *p* = 0.020), Hypoalbuminemia (OR =2.749 CI = 1.555–4.861, *p* = 0.001) and Operative time (OR =3.023, CI = 2.289–3.994, *p* = 0.001).

Univariate logistic regression analysis showed that gender (*p* = 0.563), smoking (*p* = 0.207), hypertension (*p* = 0.793), diabetes mellitus (*p* = 0.652), anaemia (*p* = 0.875), previous TURBt (*p* = 0.729), chemical therapy (*p* = 0.551), surgical approach (*p* = 0.547), estimated blood loss (*p* = 0.732) and pathological stage (*p* = 0.485) were not statistically significant factors, and therefore were not included in subsequent statistical analyses. Multivariate logistic regression analysis was performed on nine risk factors including age, BMI, constipation, renal inadequacy, previous abdominal surgery, hypoalbuminemia, urinary diversion, PLND, blood transfusion and operative time (Table 2 and Figure 1).

**Table 2.** Logistic regression assessing risk factors for POI.

Factor	Univariable analysis			Multivariable analysis		
	OR	95%CI	*p* value	OR	95%CI	*p* value
Gender						NI*
Male	1.170	0.660–2.073	0.653			
Feamle	Ref	Ref	Ref			
Smoking						NI
Yes	1.342	0.854–2.108	0.207			
No	Ref	Ref	Ref			
Hypertension						NI
Yes	0.918	0.542–1.552	0.793			
No	Ref	Ref	Ref			
Diabetes mellitus						NI
Yes	0.845	0.457–1.561	0.652			
No	Ref	Ref	Ref			
Anemia						NI
Yes	1.071	0.583–1.968	0.875			
No	Ref	Ref	Ref			
TURBt						NI
	1.097	0.698–1.726	0.729			
	Ref	Ref	Ref			
Chemical therapy						NI
Yes	1.167	0.735–1.854	0.551			
No	Ref	Ref	Ref			
surgical approach						NI
LRC	1.165	0.730–1.860	0.547			
RARC	Ref	Ref	Ref			
estimated blood loss	–	–	0.732			NI
pathological stage						NI
I	Ref	Ref	Ref			
II	2.571	0.756–8.751	0.158			
III	2.475	0.686–8.926	0.155			
IV	2.520	0.587–10.827	0.304			
intestinal tract reconstruction time	–	–	0.764			NI
Age				2.090	1.321-3.305	0.002
<65	Ref	Ref	Ref			
≥65	2.090	1.321–3.305	0.002			
BMI						
<18.5	2.500	1.070–5.840	0.038	3.021	1.760–5.186	0.001
18.5∼30	Ref	Ref	Ref			
>30	3.021	1.760–5.186	0.001	1.581	1.035–2.417	0.034
Constipation						
Yes	1.843	1.101–3.088	0.023	1.843	1.101–3.088	0.020
No	Ref	Ref	Ref			
Renal inadequacy						
Yes	1.741	0.846–3.580	0.150	1.741	0.846–3.580	0.132
No	Ref	Ref	Ref			
previous Abdominal surgery						
Yes	1.773	0.966–3.254	0.086	2.085	1.009–4.310	0.073
No	Ref	Ref	Ref			
Hypoalbuminemia						
Yes	2.632	1.507–4.600	0.001	2.749	1.555–4.861	0.001
No	Ref	Ref	Ref			
urinary diversion						
ileal conduit*	Ref	Ref	Ref			
ileal neobladder	1.493	0.904–2.466	0.139	1.580	0.942–2.652	0.083
PLND						
Yes	1.952	0.461–1.140	0.165	0.703	0.441–1.119	0.138
No	Ref	Ref	Ref			
Blood transfusion						
Yes	1.471	0.933–2.320	0.100	1.471	0.933–2.320	0.097
No	Ref	Ref	Ref			
Operative time	–	–	0.001	3.023	2.289–3.994	0.001

* :NI: Not include.

In the multivariate logistic regression model, six independent risk factors for POI were identified, including age > 65.0 years (OR = 2.090, CI = 1.321–3.305, *p* = 0.002), low body mass index (OR = 3.021, CI = 1.760–5.186, *p* = 0.001), high body mass index (OR = 1.581, CI = 1.035–2.417, *p* = 0.034), constipation (OR = 1.843, CI = 1.101–3.088, *p* = 0.020), hypoalbuminemia (OR = 2.749 CI = 1.555–4.861, *p* = 0.001) and operative time (OR = 3.023, CI = 2.289–3.994, *p* = 0.001). The remaining factors did not show significant statistical significance (Table 2 and Figure 1).

**Figure 2.**
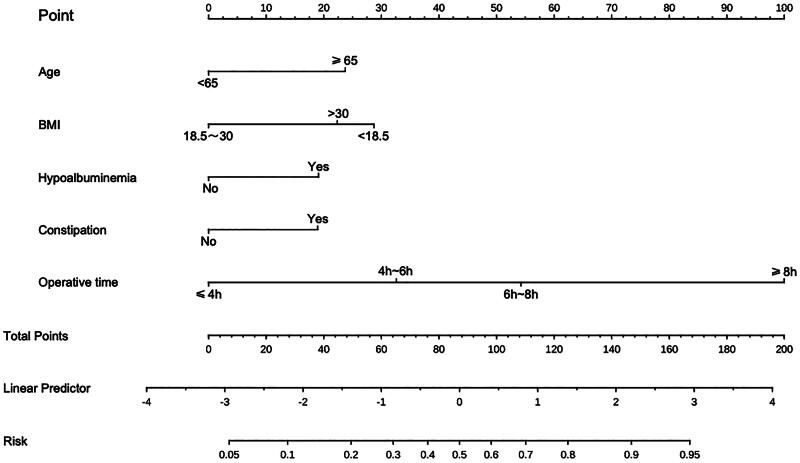
A nomogram model was established using independent risk factors screened out by multivariate regression analysis. The corresponding score for each factor is based on the condition of the patient, which can be determined by making a vertical line upwards (e.g. a patient with Hypoalbuminemia will receive 20 scores). Add all the scores to get the total score, then find the corresponding point on the total points axis and make a vertical line down to predict the risk of the POI after radical cystectomy.

### 3.3. Establishment and validation of the nomogram

A nomogram prediction model was established based on the six independent risk factors identified by multivariate logistic regression analysis (Figure 2). The predictive factor’s weight was expressed by the length of the line segment. Each risk factor was assessed individually for each patient in accordance with the circumstances, and the numbers were then summed to provide a total score. The final total score was used to determine the estimated risk likelihood of postoperative POI for each patient. The nomogram demonstrated a correlation between a higher patient score and a higher probability of POI.

**Figure 3.**
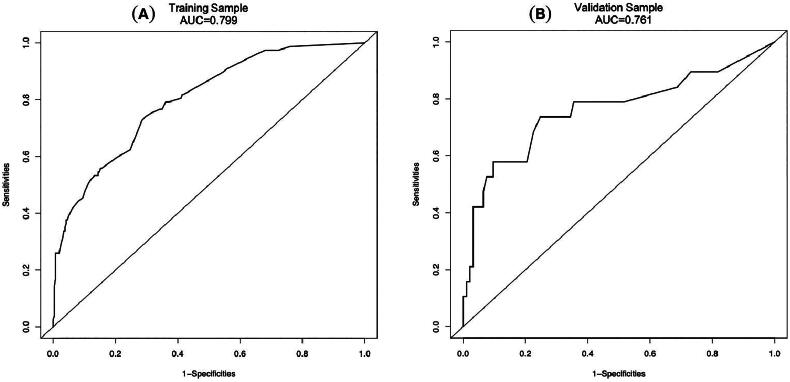
The AUC of training sample (A) and validation sample (B) showed that the model had a high discrimination ability.

**Figure 4.**
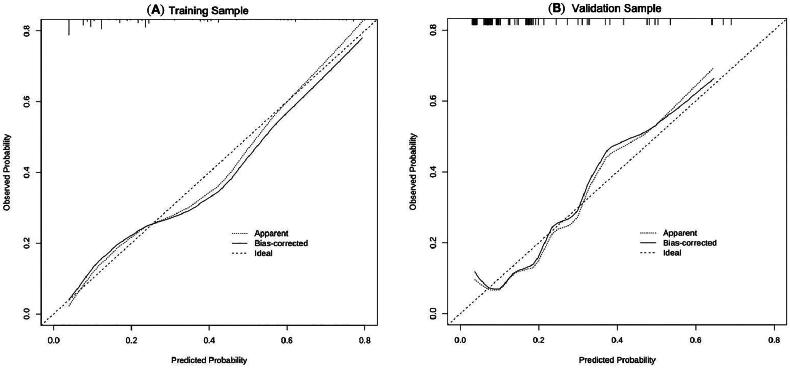
Calibration curves for training sample (A) and validation sample (B). The calibration curves for assessing the consistency between the predicted and the actual risk of POI. Favourable consistencies between the predicted and the actual risk evaluation are presented.

**Figure 5.**
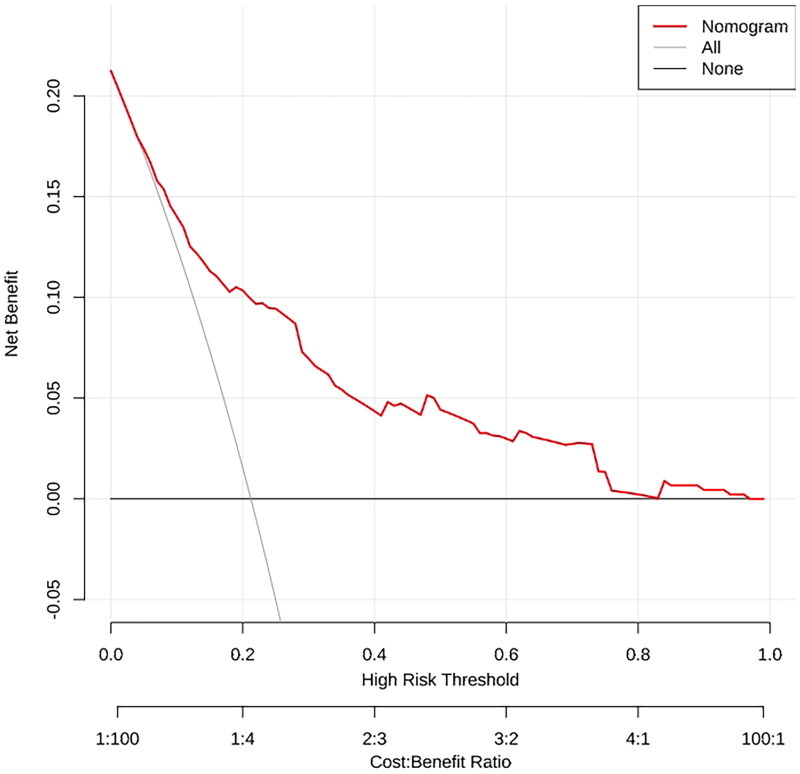
Decision Curve analysis for assessing the clinical usefulness of the nomogram. In this analysis, the decision curve provides a large net benefit across the range of 5.0% and 95.0%.

In order to determine the discrimination of the prediction model, ROC curves were drawn in the training and validation samples, and the AUC was computed. Our findings demonstrated that the model has a strong capacity for discrimination (Figure 3). The AUCs for the training samples was 0.799, while for the validation samples it was 0.761. The calibration curves were also created to display the relationship between the expected and actual values. The training and validation samples both demonstrated a good correlation between the nomogram’s projected probability and the actual circumstance (Figure 4). Besides, DCA curves were used to evaluate the clinical benefit of the nomogram model in predicting POI. When the threshold probabilities of the nomogram model were in the range of 0.05–0.90, the net benefit ratio was > 0, which suggested that the model had a better clinical value for predicting POI (Figure 5).

## 4. Discussion

POI is a common complication of RC with recently reported rates as high as 26.9% [3]. The pathophysiology of POI is still under investigation, and complex interactions between neural reflexes, inflammation, fluid overload, electrolytes, and clinical anaesthesia are thought to be involved in the development of POI [16,17]. Current studies are limited, since they are not only based on a single RC surgical approach, but also lack the prediction model of POI in patients with RC. Since the development of POI can seriously affect the recovery of patients, urologists need a predictive model to assess patient risk and make appropriate interventions. Therefore, the purpose of this study was to identify independent risk factors for POI after various RC surgical approaches and establish a user-friendly predictive model.

Several studies have demonstrated that increased patient age is associated with a higher risk of POI after RC. Robert et al. retrospectively studied 283 patients who underwent RC with pelvic nodal dissection, and using multivariate logistic regression analysis showed that the independent risk factors included increasing age [18]. One possible explanation may be the significant reductions in the number of intestinal myenteric neurons and the intrinsic and extrinsic innervation of the colorectum by visceral afferent and sympathetic motor neurons observed in advanced age patients. Furthermore, elderly patients tend to have comorbidities such as diabetes mellitus, malignant tumours, medications, and previous surgical procedures that can damage the gastrointestinal neuromuscular system [19]. In addition, since basic metabolism slows down with increasing age, a longer period of body residue of anaesthetic drugs (such as opioids) may inhibit the recovery of bowel function [20].

High BMI is related to the occurrence of POI. A recent national database found that increasing grades of obesity were independently associated with higher 30-day complication rates [21]. Robert et al. reported that 30.3% of patients with class II-III obesity were found to have POI with a hazard ratio of 1.09 [18], and proposed that obese patients may have less space for surgical manipulation leading to more obvious postoperative edoema with intestinal mucosal paralysis. In addition, a different study found that low BMI was still statistically significant [22]. This may be because patients with low BMI generally do not tolerate RC and are often malnourished, which can lead to slow recovery of postoperative gastrointestinal function.

Chronic constipation is associated with a high risk of POI. A recent study found that constipation was strongly associated with worse bowel cleanliness [23]. For patients with constipation, inadequate preoperative bowel cleansing may lead to excessive bowel preparation, which has been confirmed as a risk factor for developing POI by Xue et al. [3]. In addition, despite the lack of robust evidence for their clinical utility, laxatives or prokinetic agents have been used in clinical practice for the treatment of POI with good effect. In a study conducted by Kim and colleagues, mosapride, commonly used as a treatment for constipation, was found to be effective in reducing the rate of POI in a guinea pig model [24].

Hypoalbuminemia has also been associated with the occurrence of POI. Ornaghi et al. found that hypoalbuminemia significantly increased the rate of postoperative complications after RC [25]. Dai et al. demonstrated that preoperative hypoalbuminemia prolonged the incidence of POI by 2.7-fold in patients undergoing colectomy [26]. For patients undergoing RC, we propose that this is due to the effects of albumin on increasing plasma colloid osmotic pressure, reducing tissue edoema, as well as promoting tissue healing. When hypoalbuminemia is present, bowel edoema and delayed anastomotic healing compromise bowel function recovery.

Several studies have examined the relationship between operative time and POI. Haeuser et al. found that the operative time was associated with short-term RC complications [27]. Furthermore, these studies indicated that surgeries lasting between 4.0 and 5.0 h had the lowest complication rates, which is consistent with our findings. Greenberg et al. found that 85 out of 261 patients developed POI after abdominal surgery, and that the median operation time for patients with and without POI were 313 min and 279 min, respectively [28]. In the current study, regression analysis indicated that the operation time was an independent prognostic factor of POI after RC. We believe that an increased surgery time contributes to increased exposure of patients to higher doses of opioids. In addition, a long operation time normally causes liquid overload, acidosis and electrolyte disorders. All of these factors affect the recovery of the gastrointestinal tract, eventually leading to POI.

There are several limitations in our study. First, despite our efforts to expand the sample size, errors resulting from selection bias and recall bias may still occur. Therefore, multicentre studies with larger sample sizes are needed in follow-up studies. Second, due to the limitations of a single-center database, perioperative management of patients may differ from other institutions. Finally, we performed internal validation but did not complete external validation. Thus, future studies will use cohorts from other institutions to confirm our findings.

## 5. Conclusion

In conclusion, in the present study, we developed a prediction model for POI, which provides surgeons with a simple method that can be used to calculate the risk of POI after RC and may help surgeons to counsel and treat patients with potential risks. Further studies are required for validation.

## Ethical approval and consent to publish

This study was approved by the research ethics committee of the Second Hospital of Tianjin Medical University (authorization number: KY2023K005). Its publication is also approved tacitly by the responsible authorities where the work was carried out. The authors have no relevant financial or non-financial interests to disclose.

## Data Availability

The raw data of this study and more information can be found upon reasonable request from the author (SXY).
